# Carbon Nanomaterials for Electro-Active Structures: A Review

**DOI:** 10.3390/polym12122946

**Published:** 2020-12-09

**Authors:** Weiguang Wang, Yanhao Hou, Dean Martinez, Darwin Kurniawan, Wei-Hung Chiang, Paulo Bartolo

**Affiliations:** 1 Department of Mechanical, Aerospace and Civil Engineering, School of Engineering, Faculty of Science and Engineering, The University of Manchester, Manchester M13 9PL, UK; yanhao.hou@manchester.ac.uk (Y.H.); paulojorge.dasilvabartolo@manchester.ac.uk (P.B.); 2 Department of Chemical Engineering, National Taiwan University of Science and Technology, Taipei E2-514, Taiwan; deanaidan.martinez@gmail.com (D.M.); jdkywt@gmail.com (D.K.); whchiang@mail.ntust.edu.tw (W.-H.C.)

**Keywords:** carbon nanotubes, electro-active scaffolds, graphene, tissue engineering

## Abstract

The use of electrically conductive materials to impart electrical properties to substrates for cell attachment proliferation and differentiation represents an important strategy in the field of tissue engineering. This paper discusses the concept of electro-active structures and their roles in tissue engineering, accelerating cell proliferation and differentiation, consequently leading to tissue regeneration. The most relevant carbon-based materials used to produce electro-active structures are presented, and their main advantages and limitations are discussed in detail. Particular emphasis is put on the electrically conductive property, material synthesis and their applications on tissue engineering. Different technologies, allowing the fabrication of two-dimensional and three-dimensional structures in a controlled way, are also presented. Finally, challenges for future research are highlighted. This review shows that electrical stimulation plays an important role in modulating the growth of different types of cells. As highlighted, carbon nanomaterials, especially graphene and carbon nanotubes, have great potential for fabricating electro-active structures due to their exceptional electrical and surface properties, opening new routes for more efficient tissue engineering approaches.

## 1. Introduction

Tissue engineering is a relatively novel discipline, aiming at improving or replacing biological tissues. The use of scaffolds, physical substrates for cell attachment, proliferation and differentiation, is the most common strategy for tissue engineering [1,2,3,4]. These scaffolds must be designed according to specific requirements to create the appropriate environment for cell attachment, proliferation and differentiation. They must be biocompatible and biodegradable (the degradation rate must match the regeneration rate of the new tissue [1,2,3,4]), with proper geometry, morphology, porosity and pore interconnectivity [1,2,3,4]. Scaffolds must have adequate mechanical properties depending on the type of tissue, appropriate surface characteristics and must be easily sterilized [1,2,3,4]. A scaffold’s capacity to stimulate cells is also another important requirement.

Electrical signals are critical physiological stimuli that strongly affect cell behavior due to the cell proliferation impact on the cell membrane potential [5,6]. Electrical stimulations can redirect the alignment of random cells [7,8]. Some types of cells are aligned perpendicular to the vector’s direction of the electric field to minimize the field gradient go through the cell. Other cells are aligned parallel to the field vectors due to the electrical stimulation that causes rearrangement of the cell cytoskeleton. Additionally, the spreading direction of cells is also affected by the electric field [9]. Some types of cells migrate toward the cathode, while others toward the anode. Electrical stimulations may also affect the recognition of electrical signals and signal transduction within individual cells, gap junction intercellular communication, role of extracellular matrix and regulation of gene expression [10]. Based on these effects, applied electrical stimulations affect not only cells’ directional migration, but also cell adhesion and differentiation, DNA synthesis and protein secretion [11,12,13,14,15]. These mechanisms can contribute to both angiogenesis and osteogenesis [16,17,18,19].

Bassett et al. presented the first evidence of the electrical stimulation impact on tissues, by investigating the effects of electric current on bone regeneration in adult dogs [20]. Tissue regeneration induced by electrical stimulation was also observed in rats with sciatic nerve injuries [21]. Other researchers also observed that electrical stimulation significantly increased the DNA synthesis of osteoblasts [22], improved the contractile behavior of engineered cardiac tissue [23] and improved both myogenic differentiation and deposition of type 1 collagen [24]. Moreover, electric fields and electrical stimulations can improve the healing, wound recovery and regeneration of damaged spin cords and nerves [25].

Polymeric tissue engineering scaffolds can be fabricated with or without the incorporation of fillers, aiming to enhance mechanical or biological. Several researchers investigated the incorporation of conductive carbon nanomaterials (e.g., graphene and carbon nanotubes) into different polymer matrices to produce tissue engineering scaffolds [26,27,28,29,30]. Due to the high electrical conductivity nature of these carbon nanomaterials, these scaffolds have great potential to be used together with electrical stimulation, functioning as electro-active scaffolds for dose-promoting tissue regeneration [31]. The incorporation of conductive materials allows the transmission of electrical signals from external sources through the cell-seeded scaffolds, without compromising their mechanical, biological and degradation behavior [32]. These electrically conductive composites consist of conductive fillers blended with nonconductive biocompatible and biodegradable materials or polymer/ceramic materials. These scaffolds can be easily processed through relatively low-cost fabrication strategies, and their mechanical and electrical properties can be easily tailored. The electrical conductive properties of these structures can be empirically described according to the following equation [33]:

(1) $$\sigma =\sigma_{0}{\left(p-p_{c}\right)}^{t}$$

where σ represents the electrical conductivity of the composite material, $\sigma_{0}$ represents the scaling factor, a proportionality constant related to the intrinsic conductivity of the filler, p represents the volume fraction of the filler, $p_{c}$ represents the percolation threshold and *t* represents the critical exponent related to the dimensionality of the conductive networks in the composite material. Composites with high $p_{c}$ present high melt viscosity and inferior mechanical properties, being also more difficult to process [34,35]. The appropriate conductivity for intracellular activity was proposed to be 10^−7^–10^−2^ S/cm, depending on tissue [36].

Different processing techniques have been explored to produce scaffolds with different dimensionalities and architectures. The so-called conventional fabrication methods produce scaffolds by using fiber bonding, gas foaming, high-pressure processing, hydrocarbon templating, liquid–liquid phase separation, melt moulding, membrane lamination, polymer or ceramic fiber composite foam, solvent casting and particulate leaching methods [37]. These methods are relatively simple, but they do not allow us to control the pore architecture and pore interconnectivity, and the produced structures present limited mechanical properties and, in some cases, residual solvents [38].

Electrospinning and additive manufacturing are other relevant fabrication techniques. Electrospinning has been widely used to fabricate electro-active structures, in which process polymer and conducting materials are dissolved in a suitable solvent, and the polymer solution is dropped via a needle [1,2]. This technology allows us to fabricate 2D membranes [39,40,41], or 3D scaffolds through dry jet-wet electrospinning, even with simultaneous coating [42,43]. Centrifugal spinning and pressured gyration can also be used for tissue engineering applications [44]. Additive manufacturing describes a group of processes that create structures by joining material in a layer by layer approach. According to the American Society for Testing and Materials, additive manufacturing comprises seven techniques: material extrusion, material jetting, binder jetting, vat photopolymerization, powder bed fusion, directed energy deposition and sheet lamination (Table 1) [45]. However, only material extrusion, material jetting, binder jetting, vat photopolymerization and powder bed fusion can be used for the fabrication of biocompatible and biodegradable scaffolds. In the field of tissue engineering, additive manufacturing is the most relevant fabrication process, as it allows us to create scaffolds with precise control of the pore size, pore shape, pore distribution and pore interconnectivity.

This paper overviews the current state-of-the-art of using carbon nanomaterials (graphene, graphene oxide and carbon nanotubes) for the fabrication of electro-active structures (e.g., 3D porous scaffolds and membranes) for tissue engineering applications. Electrical properties and synthesis methods of these carbon nanomaterials are presented. A wide range of fabrication techniques are considered, and several tissue engineering applications are discussed in detail. Future perspectives and developments are also presented.

## 2. Carbon Nanomaterials for Electro-Active Scaffolds

**Figure 1:**
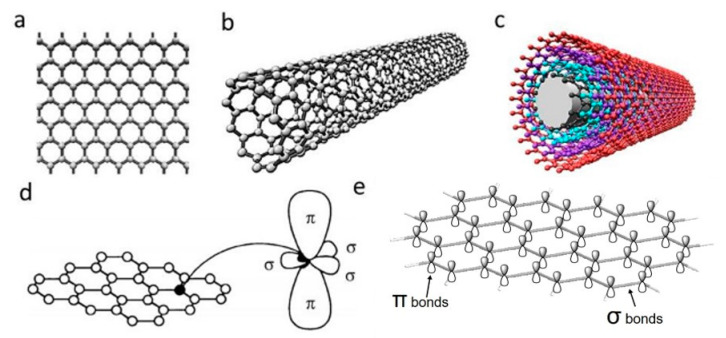
Schematic illustrations of the structures of (**a**) graphene, (**b**) single-walled carbon nanotube and (**c**) multi-walled carbon nanotube. (**d**) σ and π orbitals in carbon sp_2_ honeycomb lattice [52]; (**e**) overlapping sigma bonds in sp_2_ array of single-layer graphene. Reproduced with permission from Jorio et al., *Advanced Materials*, published by Wiley-VCH, 2011.

Carbon nanomaterials (CNMs) exhibit vast structural diversity, owing to carbon atom’s capability of covalently bonding at diverse hybridization states (sp, sp^2^ and sp^3^) with other carbon atoms and non-metallic elements [46]. The resulting allotropes are classified according to the number of dimensions, i.e., 0D, 1D and 2D, with known models such as quantum dots, nanotubes and graphene, respectively [47]. The electrical properties of carbon are highly influenced by the nanostructure anisotropy and its degree of replication [48]. All sp^2^ carbon materials are intrinsically anisotropic as it contains delocalized last non-hybridized valence π-electrons in a plane perpendicular to its basal plane. The mobility within the lattice and the dynamics in one particular configuration create “electronic layers”, responsible for the high 2D electric conductance [49]. CNMs are of similar size-scale to biological molecules, and thus can be effective platforms for enhancing biological activities within living organisms. Specifically, high surface area-to-mass ratio CNMs such as graphene and carbon nanotubes (CNTs) (Figure 1a–c), maximize the scaffold potential for cellular development, interacting with biomolecules such as DNA, enzymes, proteins and peptides [50,51].

### 2.1. Graphene

Discovered in 2004, single-layer graphene is an atomically thin film of carbon atoms bonded together in a planar 2D structure. As illustrated in Figure 1d,e, each carbon atoms are sp^2^ (planar) hybridized having covalent σ bonds with three nearest carbon atoms, forming a robust honeycomb lattice. This makes graphene currently the strongest known material with Young’s modulus of ~1.0 TPa [53]. Moreover, the exceptional light absorption properties make graphene a promising candidate for phototransistors with high responsivity and sensitivity [54].

#### 2.1.1. Electrical Properties

The ambipolar field effect on few-layers graphene, which corresponds to the availability of carriers to be tuned continuously between holes and electrons by supplying the required gate bias, was first observed by Novoselov et al. [55]. For positive gate bias, the Fermi level rises above the Dirac point, hence promoting electrons into the conduction band. On the contrary, the Fermi level drops below the Dirac point under negative gate bias, thus introducing holes into the valence band [56].

Besides the ambipolar field effect, graphene also shows the quantum Hall effect (QHE) and an extremely high carrier mobility [57,58,59,60,61,62,63,64]. As a 2D material with zero bandgaps, the electrons in graphene will be confined, leading to a quantum mechanically enhanced transport phenomena, known as QHE. However, the QHE in graphene is half-integer QHE instead of integer QHE, which is different than what is usually observed in conventional semiconductors [57]. This difference is attributed to the unique electronic properties of graphene that exhibits electron-hole degeneracy and massless Dirac fermions [57,65]. The observable QHE even at room temperature further indicates the extreme electronic quality of graphene [66].

This extraordinary electronic property is caused by the high quality of its 2D crystal lattice. In other words, graphene with higher defects density will have lower carrier mobility, since these defects act as the scattering centers, which inhibit charge transport [56]. Perpendicular to the graphene plane are the π-bonds that form delocalized electron states across the plane. Due to the easy movement of electrons in these π-states, high carrier mobility of ~200,000 cm^2^ V^−1^s^−1^ has been attained for suspended graphene and ~500,000 cm^2^ V^−1^s^−1^ for graphene-based field-effect transistor [63,64,67]. Consequently, the charge transport at such high value of carrier mobility is essentially ballistic on the micrometer scale, at room temperature [56], making graphene a useful material for biosensing and biomedical applications [68].

#### 2.1.2. Materials Synthesis

**Figure 2:**
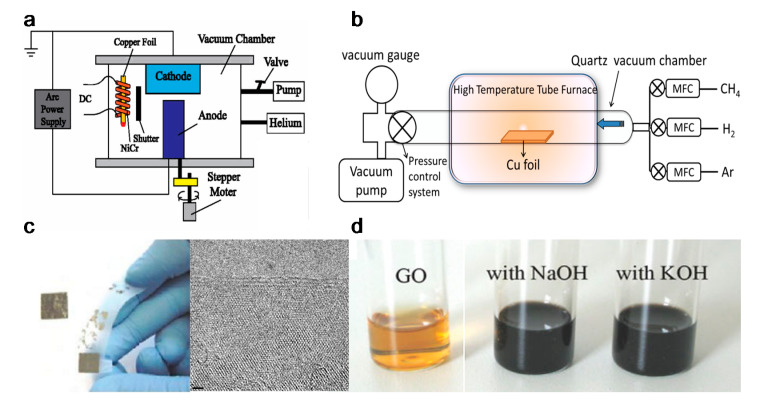
Graphene top-down synthesis methods. Schematic of (**a**) arc discharge [75] and (**b**) chemical vapour deposition (CVD) setup [76]; (**c**) micromechanical exfoliation of graphite [77] and TEM image [78]; (**d**) the deoxygenation of exfoliated graphene oxide (GO) under alkaline conditions [79]. Reproduced with permission from Fan et al., *Journal of Applied Physics*, published by American Institute of Physics, 2015; Kumar and Lee, *Advances in Graphene Science*, published by Books on Demand, 2013; Singh et al., *Progress in Materials Science*, published by Elsevier, 2011; Meyer et al., *Nature*, published by Nature, 2007; and Fan et al., *Advanced Materials*, published by Wiley-VCH, 2008.

Graphene can be produced by using top-down and bottom-up synthesis methods. Top-down synthesis methods of graphene are generally detachment or exfoliation from existing graphite crystals [69]. Exfoliation can be done mechanically (Scotch Tape method) [70], in liquid phase, exploiting ultrasounds to graphite or graphite oxide sheets by using chemicals with matching surface energy [67,71,72,73], or by electrical arc-discharge between two graphitic electrodes (Figure 2) [74].

Mechanical exfoliation (repeated peeling), the first reported approach for graphene fabrication, was initially described by Novoselov et al. [55]. Moreover, the electrical field effect of single-layered graphene from the mechanical exfoliation of small mesas of highly oriented pyrolytic graphite was also observed [55].

Liquid-phase exfoliation (LPE) is another synthesis method being characterized by its low cost, ease of operation and minimal environmental impact. Manna et al. demonstrated single- and few-layers of graphene nanosheets synthesis from bulk materials by a surfactant-free LPE, using water as the co-solvent with *N*-methylpyrrolidinone (NMP) [73]. Authors proved that interactions in both solid–solvent and solvent–solvent interactions could influence the LPE process [72]. Layered-materials (solid) and solvent system (liquid) interaction improves the exfoliation efficiency by minimizing solid–liquid interfacial energy (*γ*_sl_), maximizing solid–liquid interfacial work of adhesion (*W*_sl_) at the optical *m*_w_. Moreover the water–NMP (liquid–liquid) heteroassociation prevents the recombination of exfoliated layers, and the bulky (NMP·2H_2_O)_n_ aggregates are able to provide intersheet repulsive forces, separating the nanosheets with non-overlapping Leonard–Jones (L–J) potentials. Briefly, 50 mg of bulk materials were placed in 14 mL centrifuge tubes with an initial concentration of 5 mg/mL for exfoliation. The materials were batch sonicated for 6 h at the power of 100 W and a frequency of 37 kHz. Every 30 min, the positions of each sample tubes were changed to achieve uniform power distribution and the water of bath sonicator was replaced to maintain the temperature between 27 and 37 °C during the sonication process. The dispersions were stored overnight and centrifuged at 3000 rpm for 30 min. According to TEM measurement, the lateral size of the exfoliated graphene was 500–2000 nm, the optimal water–NMP mixed solvent mass fraction was 0.2–0.3, which result to 0.43 (~8.6% by mass) mg/mL of exfoliated graphene nanosheets [72]. However, limited scalability, controllability and size of graphene or other 2D materials are the main limitations in the LPE process [80].

Oxidation-reduction (redox) is another top-down synthesis method. GOs produced by Hummers method can be reduced into graphene with different kinds of reducing agents, such as N_2_H_4_ and NaBH_4_ [80,81]. Nevertheless, the Hummers method suffers from some drawbacks, including high oxidants consumption, inevitable intercalating agents, long process time, high cost and poor scalability [82]. Schniepp et al. utilized a different approach to produce single layer graphene sheets [83], based on a redox method combined with thermal treatment, which mainly attributed to the interstices between the graphene sheets due to the CO_2_ expansion during rapid heating of GOs. Therefore, complete graphite oxidation and extremely rapid heating of GOs are fundamentally required. Briefly, natural flake graphite was oxidized in a mixture solution of sulfuric acid, nitric acid and KClO_3_ for more than 96 h. After the 0.34 nm intergraphene spacing disappears, and a new spacing of 0.65–0.75 nm range appears (depend on GOs water content), the GOs are dried and purged with argon for thermal exfoliation. The rapid heating rate of 2000 °C/min to 1050 °C would split the GOs into several individual sheets through CO_2_ evolution. Successful exfoliation was confirmed when all diffraction peaks were eliminated. Atomic force microscopy (AFM) measurements show that the produced graphene sheets are well dispersed at an average density of about 50 flakes per 100 μm^2^ and exhibit a lateral extent of a few hundred nanometers. The representative height varies at two length scales, 2 nm for the flat areas with respect to HOPG and 10 nm for the several large, meandering wrinkles [83].

On the other hand, bottom-up synthesis deal with directly growing graphene layers on substrate surfaces. This method includes epitaxial growth on silicon carbide crystal and chemical vapour deposition (CVD) where graphene from a hydrocarbon source precipitates from the transition metal surface [84,85]. Synthesis through CVD is the most viable method in terms of operational control, complexity and throughput [69].

**Figure 3:**
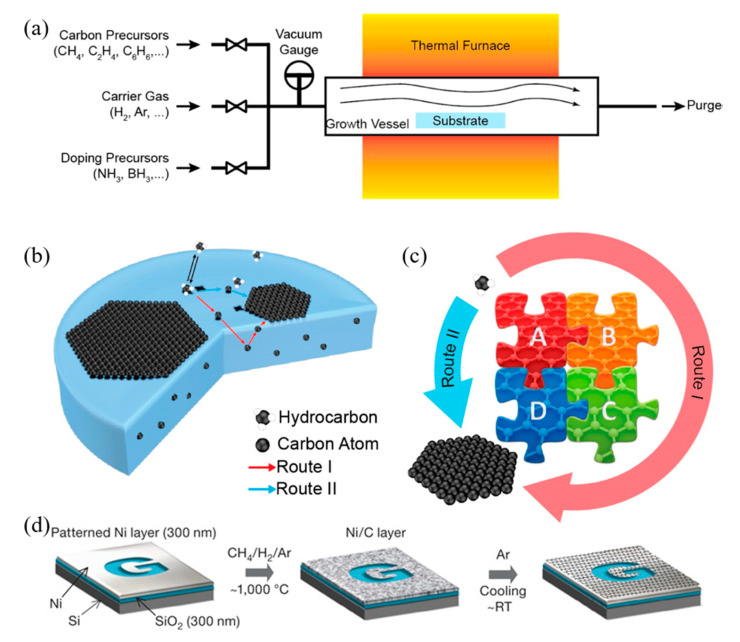
(**a**) Sketch drawing of typical CVD system for graphene fabrication; (**b**) elementary steps involved in CVD process (red arrow represents good metal to carbon affinity, while blue arrow represents poor metal to carbon affinity); (**c**) schematic illustration of four elementary steps connected together and coexistence of two routes for carbon precursors conversion to graphene [88]; (**d**) example of CVD synthesis of patterned graphene films on thin nickel films [89]. Reproduced with permission from Yan et al., *Accounts of Chemical Research*, published by American Chemical Society, 2013; and Kim et al., *Nature*, published by Nature, 2009.

Due to the ease of controllability and scalability, graphene films with large area and high quality can be obtained via CVD process [86]. Figure 3a–c shows a typical CVD process, which involves the deposition of volatile precursors on the exposed substrate surface to produce the desired graphene or 2D materials films. Depending on the substrate’s catalytic ability, the growth of graphene is governed by two instances: heterogeneous catalysis (governs the growth process for substrates with high catalytic ability) and gas reaction (governs the growth process for substrates with low catalytic ability). Heterogeneous catalysis is more suitable for high-quality graphene films fabrication [87]. Therefore, the key parameters in the CVD process are the catalyst, precursor, flow rate, temperature, pressure and time.

The graphene growth on the metal substrate based on heterogeneous catalysis CVD process consists of four steps:

1. Adsorption and catalytic decomposition of precursor gas.
2. Diffusion and dissolution of decomposed carbon species on the surface and into the bulk metal.
3. Segregation of dissolved carbon atoms onto the metal surface.
4. Surface nucleation and growth of graphene.

Another different route occurs for metal with poor carbon affinity (e.g., Cu), in which the decomposition of carbon precursors was directly followed by graphene formation, realized by diffusion of carbon atoms on the metal surface. These two routes coexist in all graphene CVD system, but dominant depends on the properties of metal substrates [88].

Somani et al. reported that few-layered graphene could be obtained by CVD synthesis on nickel sheets [90]. Similarly, Kim et al. reported that graphene obtained by CVD synthesis on thin nickel films yielded good electronic properties comparable to exfoliated graphene. Briefly, as shown in Figure 3d, an electron-beam evaporator deposit thin layers of nickel with a thickness larger than 300 nm on SiO_2_/Si substrates. The samples were heated to 1000 °C in a quartz tube under an argon atmosphere. After flowing the reaction gas mixtures (CH_4_:H_2_:Ar = 50:65:200 standard cubic centimeters per minute (sccm) ), the samples were rapidly cooled to room temperature (~10 °C/s), using flowing argon, which is essential to prevent the multi-layers formation and efficiently separate graphene layers in the later process [89]. Li et al. also utilized copper foils as a catalytic substrate to improve graphene layer homogeneity with >95% consisted of a single layer [91].

Different synthesis methods significantly affect the properties of graphene such as surface area, number of layers, lateral dimension, surface chemistry, hydrophilicity and purity. These parameters also have an impact on the biological effects of graphene [92]. It is reported that with the decrease of lateral size of graphene nanosheets, the viability of bacteria is also decreased [93]. Besides the C/O ratio (for GO), structural defects, dopants and metallic residues also influence the biological properties of the produced graphene scaffolds [94,95].

#### 2.1.3. Tissue Engineering Applications

**Figure 4:**
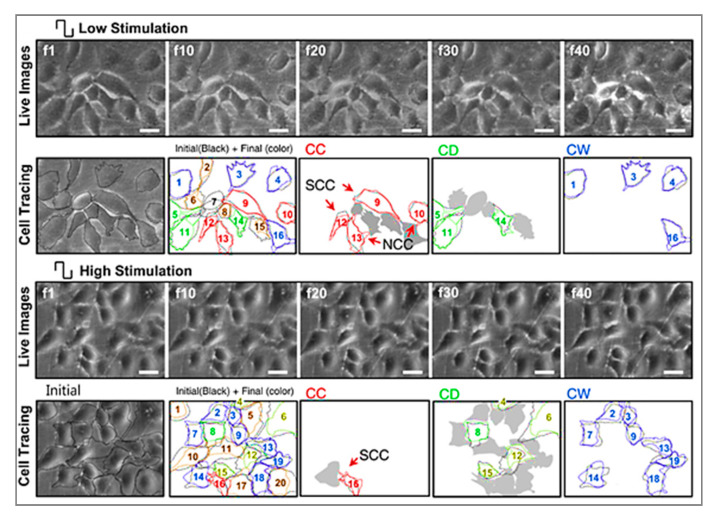
Representative images and analysis of cellular response to electrical stimulation; “f1” to “f40” corresponds to the 1st to 40th image taken during stimulation, using an optical microscope; “f1” cell shapes are outlined in black. The final shapes are then represented by different colors (red for cell-to-cell coupling (CC), green for cell-to-cell decoupling (CD) and blue for cell-to-cell wavering (CW)). (Top, low stimulation) Stimulation at 4.5 mV/mm where CC categorization was observed in the majority of the cells, also with clear newly formed cell-to-cell coupling (NCC) and strengthened cell-to-cell coupling (SCC). (Bottom, high stimulation) Stimulation at 450 mV/mm, where CD and CW categorized cells are more evident. Scale bar represents 30 mm [101]. Reproduced with permission from Heo et al., *Biomaterials*, published by Elsevier, 2011.

After production, graphene can be reformed into zero-dimensional nanomaterial, rolled into one-dimensional nanotube or manipulated into 3D graphite [51]. Dispersed graphene and graphene oxide (GO) and its interaction with target cells have been explored [96,97,98]. Multiple reports have indicated that graphene is an outstanding platform for promoting the adhesion, proliferation and differentiation of different cell types, such as mesenchymal stem cells (MSCs), neural stem cells (NSCs), embryonic stem cells (ESCs) and induced pluripotent stem cells (iPSCs) [99,100,101,102]. In the case of neural cells, graphene was found to be capable of forming a functional neural network as demonstrated by Serrano et al. where GO 3D scaffolds were fabricated through a biocompatible freeze-casting process named ice segregation-induced self-assembly (ISISA) [103]. Positive results, such as improved neural network interconnection and an increase in dendrites, axons and synaptic connections, were observed. Graphene also has great potential for neural interfacing, promoting the neurite sprouting and outgrowth of hippocampal neurons in primary culture [100]. Heo et al. investigated neural cell-to-cell interactive reactions on graphene/poly (ethylene terephthalate) films with SHSY5Y human neuroblastoma cells, followed by electrical stimulation at low and high magnitude [101]. As shown in Figure 4, cell-to-cell interactions can be classified into either cell-to-cell decoupling (CD) or cell-to-cell coupling (CC). Furthermore, the CC group can be divided into newly formed cell-to-cell coupling (NCC) and strengthened cell-to-cell coupling (SCC). Cell-to-cell wavering (CW) was also covered. Low electrical field stimulation (4.5 mV/mm), resulted in the highest percentage of CC effect, including NCC and SCC. With high electrical field stimulation (450 mV/mm), the main reaction of cells was CD and CW. These results show that cell-to-cell decoupling is enhanced under high stimulation, while non-contact weak electric field stimulation also enables cell-to-cell coupling without cellular death [101].

**Figure 5:**
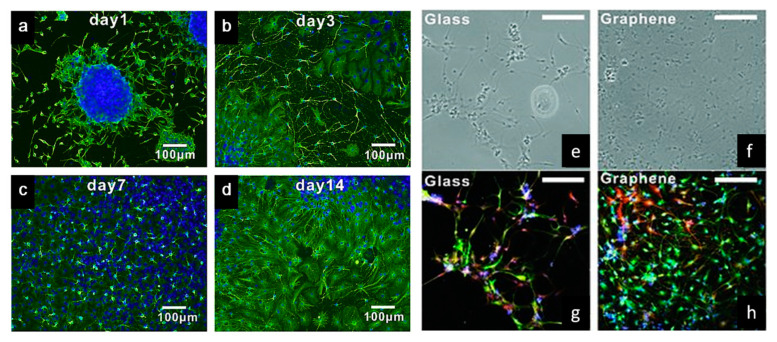
(**a**–**d**) Immunostaining (B-tubulin) of hNSCs differentiation developing neural networks on graphene substrates [104]. (**e**,**f**) Bright-field and (**g**,**h**) fluorescence microscopy images of immunostained differentiated hNSCs on glass and graphene substrates, after one month of cell culture. DAPI (blue) for nuclei, TUJ1 (green) for neural cells and GFAP (red) for astroglial cells [105]. Reproduced with permission from Tang et al., *Biomaterials*, published by Elsevier, 2013; and Park et al., *Advanced Materials*, published by Wiley-VCH, 2011.

Tang et al. examined the development of neural network from human neural stem cells (hNSCs) differentiation at graphene by comparing fluorescence images from day 1 to day 14 [104]. After seeding, it was possible to observe that the cells were able to adhere to the substrates. As indicated in Figure 5a–d, one day after cell seeding, cells are able to migrate, to different directions from neurospheres. After 14 days, high portions of the neurites contacted each other resulting in subsequent synapse formation. A study comparing cell differentiation on glass and graphene substrates was also conducted by Feng et al. [102]. As shown in Figure 5e–h, after one month, higher hNSCs adhesion and differentiation were observed with graphene substrate. The results show that the differentiation of hNSCs more toward to neuron than glial cells, and graphene functioned as a good cell adhesion layer during the long term differentiation process [105].

Jakus et al. prepared a custom-sized nerve conduit based on graphene and poly (lactide-co-glycolide) (PLG), using an extrusion-based additive manufacturing technology [106]. Results show that by increasing the graphene concentration from 20 vol.% (or ~32 wt.%) to 60 vol.% (or ~75 wt.%), strain decreased from 210% to 81%, and conductivity increased from 200 to 600 S/m, increasing also hMSCs proliferation. Moreover, it was also observed that the expressions of certain neuronal-specific markers such as glial fibrillary acidic protein (GFAP), neuron-specific class III *β*-tubulin (Tuj1) and microtubule-associated protein 2 (MAP2) significantly increased after 14 days of cell differentiation [106].

**Figure 6:**
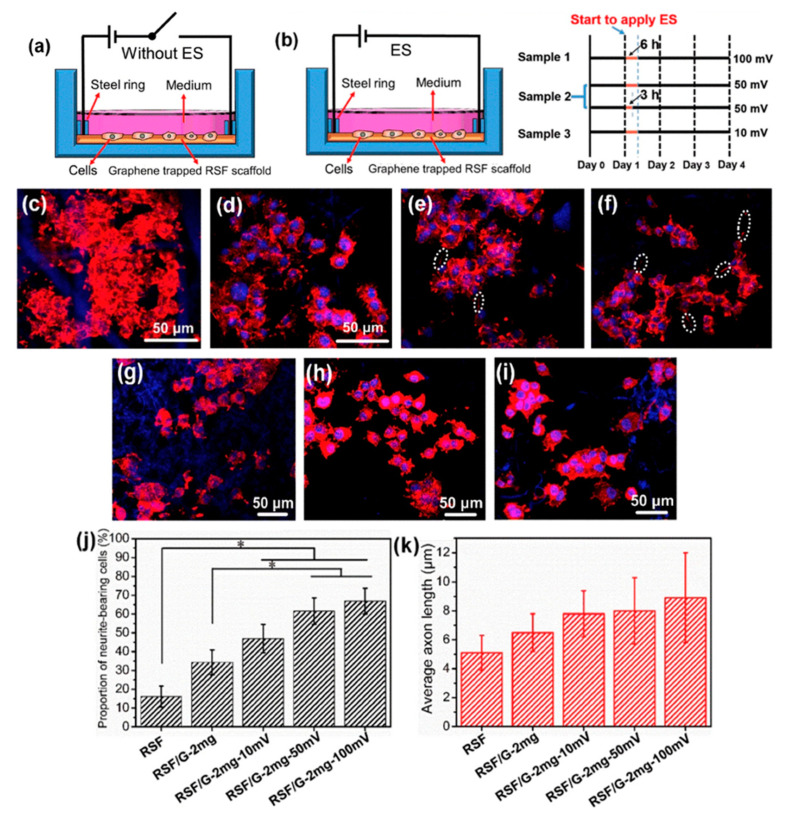
(**a**,**b**) Schematic representation of the cell culture device (**a**) without electrical stimulation (ES) and (**b**) with ES. The right-hand side of (**b**) shows the ES experimental design, where black lines indicate periods without ES, and yellow lines indicate periods with ES. (**c**–**f**) Representative laser scanning confocal microscope images of PC12 cells cultured on (**c**) regenerated silk fibroin (RSF), (**d**) RSF/G-1mg, (**e**) RSF/G-2mg and (**f**) RSF/G-4mg for four days, without ES (white ellipses indicate axons). (**g**–**j**) Laser scanning confocal microscope images of PC12 cells cultured on RSF/G-2mg scaffolds for four days with voltages of (**g**) 10 mV, (**h**) 50 mV and (**i**) 100 mV. (**j**) The proportion of PC12 neurite-bearing cells. (**k**) Average axon length of PC12 cells with and without ES (* *p* < 0.05). (**g**–**k**) ES time is 6 h [107]. Reproduced with permission from Zhang et al., *Carbon*, published by Elsevier, 2019.

Zhang et al. developed an approach which successfully added graphene into regenerated silk fibroin (RSF) scaffolds. As Figure 6 shows, biological evaluation of SCs and PC12 cells shows that the fabricated scaffolds, with the lowest resistance of 54.9 ± 20.3 Ω/sq., can effectively promote the attachment, proliferation and differentiation of the cells. The neurite growth of PC12 cells can also be simulated by the scaffolds [107]. Zhao et al. [108] and Yang et al. [109] also evaluated the graphene/silk fibroin (SF) conductive fibrous scaffolds fabricated by electrospinning. The results show that scaffolds with higher graphene concentrations exhibited higher currents and thus, higher conductivity [108]. However, the graphene concentration higher than 3 wt.% shows negative effects on cell proliferation [109].

**Figure 7:**
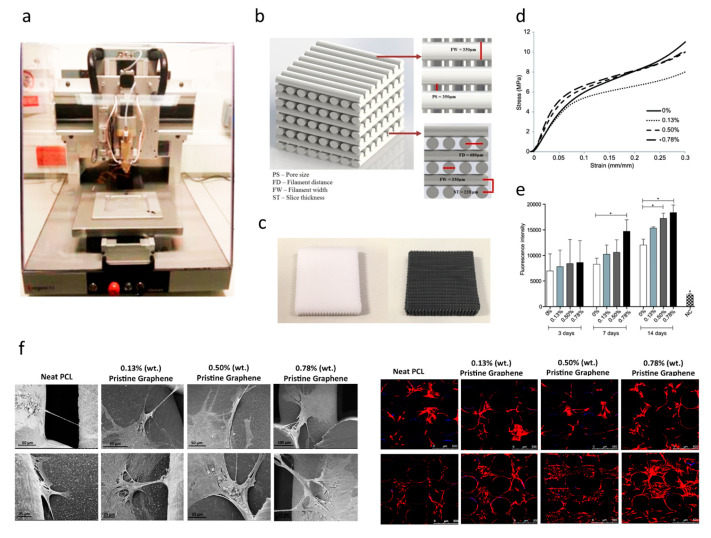
(**a**) Extrusion-based additive manufacturing system; (**b**) design of the PCL and PCL/ graphene scaffolds; (**c**) PCL and PCL/graphene scaffolds after fabrication; (**d**) mechanical characterization; (**e**) biological characterization (Alamar Blue assay); (**f**) scanning electron microscopy (SEM, left) and confocal microscopy images (right) of cell-seeded scaffolds [110,111,112]. Reproduced with permission from Wang et al., *International Journal of Bioprinting*, published by Whioce Publishing Pte. Ltd., 2016; *Materials*, published by MDPI, 2016; and *2nd International Conference on Progress in Additive Manufacturing*, published by Research Publishing, 2016.

For bone tissue engineering applications, Wang et al. [110,111,112,113] explored the use of an extrusion-based additive manufacturing system to produce poly(*ε*-caprolactone) (PCL)/graphene scaffolds. The effect of adding graphene to the polymeric scaffolds was studied form a morphological, physiochemical and biological point (Figure 7). Results show that the addition of small quantities of graphene has a positive impact in terms of mechanical properties, cytocompatibility and stimulating cell proliferation. PCL/graphene scaffolds with a squared pore size of 350 µm were produced by using a screw-assisted extrusion additive manufacturing system. The results show that by increasing the graphene content from 0 to 0.78 wt.%, the compression modulus increased from 82.2 ± 6.8 MPa to 128.7 ± 6.9 MPa. Cell proliferation of human adipose-derived stem cells (hADSCs) was also significantly increased due to the presence of graphene. Other studies also show that graphene can be used to accelerate the osteogenic differentiation of hADSCs [114,115].

**Figure 8:**
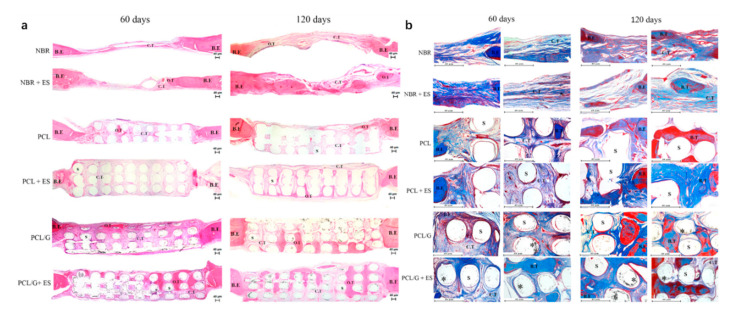
Photomicrography of the defect area after 60 and 120 days of in vivo bone regeneration test. (**a**) Attained with hematoxylin and eosin at 50× *g* magnification. (**b**) Stained with Masson Trichrome at 100× *g* magnification showing areas of the bone defect. In these images, it is possible to observe the bone edge (B.E), scaffold (S), connective tissue (C.T), bone tissue (B.T), graphene nanosheets (*) and matured/organized tissue (O.T) [32]. Reproduced with permission from Wang et al., *Materials Science and Engineering: C*, published by Elsevier, 2019.

Further in vivo investigations were conducted based on a male Wistar rats’ model [32,116]. Six testing groups were considered: NBR (natural bone regeneration), NBR+ES (natural bone regeneration with electrical stimulation), PCL (PCL scaffolds), PCL+ES (PCL scaffolds with electrical stimulation), PCL/G (PCL composite scaffolds containing 0.78 wt.% of graphene) and PCL/G+ES group (PCL composite scaffolds containing 0.78 wt.% of graphene with electrical stimulation) as shown in Figure 8. Results show that the scaffold-based strategy, especially scaffolds containing graphene and combined with electrical stimulation, present better results in terms of bone regeneration than the natural bone repair (NBR) group. After 60 days of implantation, scaffolds containing graphene promoted higher connective tissue formation and bone mineralized tissue formation than NBR group and PCL group. Additionally, PCL+ES (31% of cumulative tissue formation), PCL/G (38.2%) and PCL/G+ES (41.2%) allowed for more new-formed tissue than the NBR group (17.6%) (Figure 8). After 120 days of implantation, the applied electrical stimulation allows for high levels of new and more organized bone formation.

**Figure 9:**
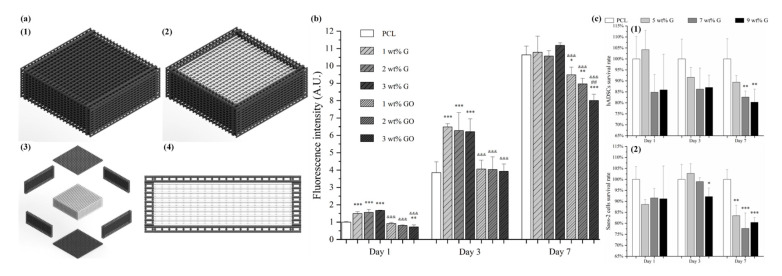
(**a**) Schematic drawing of dual-functional scaffolds including overall view (with (a1) and without top (a2), exploded view (a3) and side view (a4)). (**b**) *In vitro* cell viability/proliferation results of PCL/graphene and PCL/GO scaffolds with hADSCs. (**c**) Comparison test on PCL/graphene scaffold using hADSCs (c1) and Saos-2 cells (c2) [117,118]. Reproduced with permission from Hou et al., *International Journal of Bioprinting*, published by Whioce Publishing Pte. Ltd., 2020; and *3D Printing and Additive Manufacturing*, published by Mary Ann Liebert Inc., 2020.

Hou et al. proposed a novel concept of dual-functional scaffold (Figure 9) for both bone cancer treatment and bone regeneration, using graphene and GO fillers [117,118]. The scaffolds were produced by using screw-assisted extrusion-based additive manufacturing system with PCL as the polymeric matrix. Experimental results showed that the addition of both graphene and GO enhances the mechanical properties of PCL scaffolds, allowing to obtain scaffolds with compressive modulus in the same order of magnitude as human trabecular bone. In vitro biological studies were conducted, using both hADSCs and bone cancer cells Saos-2. Results show that scaffolds with GO fillers showed greater inhibition ability than scaffolds with graphene fillers. Furthermore, scaffolds containing high dose (5, 7 and 9 wt.%) of graphene showed greater inhibition ability on Saos-2 cells than hADSCs.

For cartilage tissue engineering applications, Liao et al. [119] fabricated scaffolds composed of chondroitin sulfate methacryloyl, poly(ethylene glycol) (PEG) methyl ether-*ε*-caprolactone-acryloyl chloride and graphene oxide (CSMA/PECA/GO), using a thermal-initiated free-radical polymerization method. In vitro biological assessments suggested that the seeded chondrocytes were able to attach proliferate. Moreover, for the in vivo biological assessment on osteochondral defects of a rabbit model, compared to the scaffold without cells, scaffolds with cell injection induced higher volume of newly formed cartilage/bone tissues [119].

Hitscherich et al. investigated the potential of PCL/graphene scaffold for cardiac tissue engineering applications. The scaffolds were prepared through electrospinning considering different graphene concentrations (0.01% and 0.5%). Electrical stimulation results show that the impedance of the scaffolds decreased by increasing the graphene contents. In vitro studies indicate that the fabricated scaffolds were biocompatible, able to support stem cell-derived cardiomyocytes, and to improve the Ca^2+^ handling properties of mouse embryonic stem cell derived cardiomyocytes (mES-CM). This can be explained by the local conductive pathways of the scaffolds which facilitated signal propagation and interaction between cells [120]. Bahrami et al. reported that three-dimensional graphene foams, produced by using a CVD method, could reach an electrical conductivity of 9 S/cm^−1^, thus stimulating a high level of the cardiac-specific genes Conx43 and TrpT-2 after seven days of cell seeding without the use of external electrical stimulation [121].

**Figure 10:**
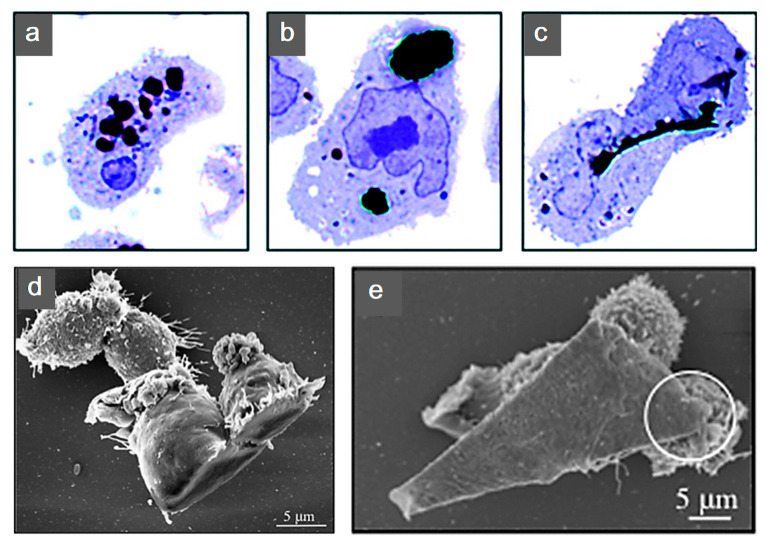
Human THP-1 macrophages internalization of few-layered graphene (FLG). Untreated cells were exposed to (**a**) 550 nm, (**b**) 800 nm and (**c**) 5 μm FLG sizes. For interaction to become more apparent under light microscopy, cells were stained with blue. (**d**,**e**) Macrophage interaction with FLG [140]. Reproduced with permission from Sanchez et al., *Chemical Research in Toxicology*, published by American Chemical Society, 2011.

Additional biological studies using graphene electro-active structures are summarized in Table 2. However, further research is still required. The cytotoxicity introduced from graphene into these substrates is still under investigation. Research reported layered graphene sheets up to 5 μm in lateral dimension can be internalized by macrophages by adhering initially, gradually spreading and covering few-layered graphene (FLG) surface, without perturbation of their plate-like shape (Figure 10). As featured by Liao et al. [97], using human erythrocytes and skin fibroblasts, further modifying size, shape and surface chemistry of graphene, can highly influence the cytotoxicity. In addition, cover the graphene surface with biocompatible polymers can be regarded as a common method to reduce the cytotoxicity, this can also improve the solubility, stability and retention time in the blood stream [98]. Furthermore, graphene cytotoxicity is also closely associated with the biocompatibility of its surface functionalization, non-functionalized counterparts were found to be more toxic [122]. However, longer term studies need to be conducted, such as preclinical studies considering different animal models [96].

### 2.2. Carbon Nanotubes

**Figure 11:**
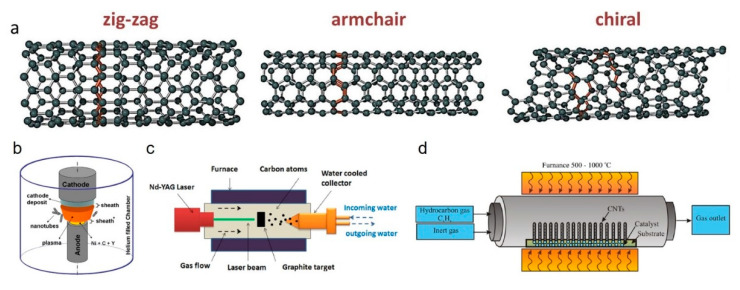
(**a**) Single-walled carbon nanotubes (SWCNTs) configuration [145]. Three common systems for carbon nanotube (CNT) synthesis: (**b**) arc-discharge [146], (**c**) laser ablation [147] and (**d**) thermal CVD [148]. Reproduced with permission from Grobert, *Materials Today*, published by Elsevier, 2007.

Carbon nanotubes (CNTs) are cylindrical tubes of sp^2^ bonded carbon atoms, conceptually regarded as rolled-up sheets of graphene [141]. CNTs are considered 1D and highly anisotropic materials as their aspect ratio (length/diameter) frequently exceed 10,000 with ends un/capped by semi-fullerene molecules (pentagonal ring defect) [46]. CNTs can be classified as single-walled carbon nanotube (SWCNT) or multi-walled carbon nanotube (MWCNT) depending on the number of concentrically arranged graphene layers [142,143]. SWCNT diameter typically ranges from 0.4 to 2 nm [46], while MWCNTs outer diameter varies from 2 to 30 nm with an interlayer spacing of 0.34–0.39 nm creating a coaxial nanotube assembly resembling a Russian-doll [144]. According to the chirality (the orientation of graphene lattice with respect to tube axis), SWCNTs are classified as armchair, zigzag and chiral (Figure 11a). Since CNTs are basically graphene in different dimensions, CNTs also present similar electrical, thermal and optical properties due to the extended sp^2^ carbon and tunable physical properties.

#### 2.2.1. Electrical Properties

Contrary to graphene, QHE does not exist in CNTs. The properties of CNTs can be tuned by controlling the diameter, length, single-walled or multi-walled, surface functionalization and chirality [149,150]. Doping CNT with heteroatoms such as nitrogen or boron is also an effective way to control its electrical properties [151], thermal properties [152] and chemical properties [153].

**Figure 12:**
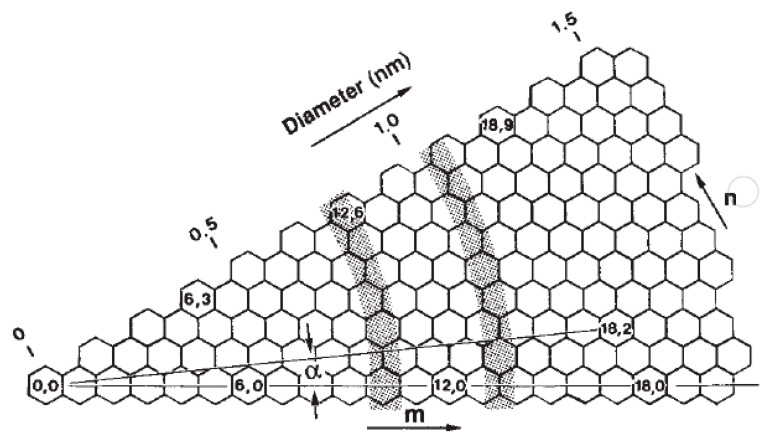
Schematic representation of different chirality indices resulted from different rolled-up vector for honeycomb graphene structure [143]. Reproduced with permission from Lijima and Ichihashi, *Nature*, published by Nature, 1993.

The structure of SWCNTs can be represented by the chirality indices (*n*,*m*), which are equivalent to the diameter (*d*) and the chiral angle (*α*) [143,154]. Figure 12 describes different rolled-up vectors of honeycomb graphene structures, resulting in different chirality indices. The *m* index is assigned to *m*th hexagon from the origin, whereas the *n* index is ascribed as *α*. Therefore, CNTs structure can be uniquely determined when the diameter and the angle are known [143]. Armchair SWCNTs exhibit a metallic behavior as they have finite density of states at Fermi level, while chiral SWCNTs exhibit a semiconductor behavior given a zero density of state of the small bandgap featured in their band structure [46]. MWCNTs are less reactive than SWCNTs because of the larger outer diameter and lower curvature [141].

#### 2.2.2. Materials Synthesis

Three different methods can be used to synthesize CNTs (MWCNTs and SWCNTs) (Figure 11b–d): CVD [155,156], carbon arc-discharge [157] and laser ablation [158]. In some methods, such as arc-discharge and laser ablation, MWCNTs are synthesized in the absence of a catalyst, and SWCNTs are synthesized in the presence of carbon electrodes containing catalytic metal particles [159]. As mentioned, the electronic properties of CNTs are strongly related to their chirality and CVD is the most widely used synthesis method, allowing large-scale production of CNTs and the control of SWCNT chirality [155,160]. CVD techniques include plasma-enhanced (PE) oxygen assisted CVD [161], microwave plasma (MPECVD) [162] and radio frequency (RF-CVD) [163]. Among all of these methods, the most powerful and standardized method is the catalytic chemical vapour deposition [46].

The key factor in controlling the chirality of SWCNTs in these CVD methods is the initial nucleation stage, in which the hemispherical cap composed of six pentagons are formed [164], and the chirality of an SWCNTs is determined [165]. Therefore, the structure of each nanotube can be defined by controlling key parameters such as catalysts [155,160,166,167,168,169,170], feedstock [171], temperatures [172], pressure [167], gas compositions [172,173] and reaction time [169]. Among these parameters, the catalysts seem to play a dominant role in obtaining specific nanotubes chirality [155].

**Figure 13:**
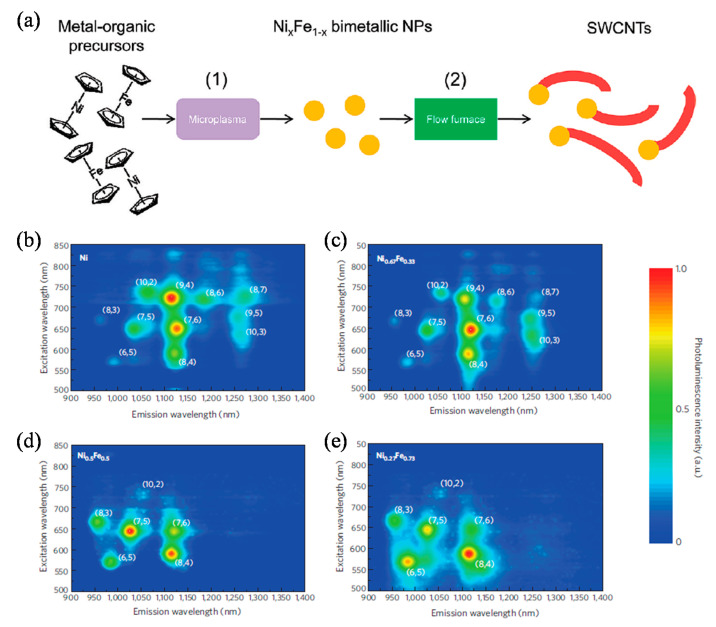
(**a**) Stepwise, a gas-phase process for SWCNTs growth consisting of (1) microplasma synthesis of compositionally tuned bimetallic nanoparticles and (2) thermal growth of CNTs [160]. Contour plots of the photoluminescence as a function of excitation and emission for SWCNTs samples grown with (**b**) Ni, (**c**) Ni_0.67_Fe_0.33_, (**d**) Ni_0.5_Fe_0.5_ and (**e**) Ni_0.27_Fe_0.73_ nanocatalysts at a constant mean particle diameter of 2.0 nm. The chiral indices are indicated as (*n*, *m*) for each corresponding peak [155]. Reproduced with permission from Chiang et al., *ACS Nano*, published by American Chemical Society, 2009; and *Nature Materials*, published by Nature Publishing Group, 2009.

Many metal catalysts such as bimetallic catalysts have been used for catalytic chiral specific CNTs growth [167,174]. However, the catalyst size and composition were hardly independently controlled. Chiang et al. [155] designed a system that consists of a two-step process, using bimetallic NiFe as the catalyst. As shown in Figure 13a, the bimetallic nanocatalysts were synthesized in a continuous-flow atmospheric pressure microplasma and nanotube growth in a tubular flow furnace. The as-synthesized nanocatalysts were introduced into a tubular flow furnace with 0.5 sccm C_2_H_2_ and 50 sccm H_2_ and heated at 600 °C. The chiral indices of the produced SWCNTs with different nanocatalysts compositions were analyzed by photoluminescence (PL) characterization (Figure 13b–e). The narrowest (*n*, *m*) distribution was obtained for SWCNTs grown with Ni_0.27_Fe_0.73_ nanocatalysts with dominating structure of (8, 4) and smaller fractions of (7, 5), (6, 5), (7,6) and (8, 3) [155].

The key point in achieving nanotube with specific chiral indices is to maximize the structural match between the nanocatalysts crystal planes and the end structure of nanotube chirality [168,169,170]. Yang et al. [170] reported several SWCNTs template synthesis with specific chirality (*n*, *m*), using W_6_Co_7_ alloy nanocatalysts. Briefly, SWCNT growth was performed in a quartz reactor with an inner diameter of 2.1 cm by using an ethanol CVD method. The precursor solution was dropped onto the SiO_2_/Si substrates and was calcined at 700 °C in an air containing tube furnace for 3 min. After purging the system with Ar, two hydrogen flows (80 cm^3^/min and then 200 cm^3^/min) were introduced through a water bubbler (ice-water bath) to reduce the calcined catalyst precursors, using a TPR method from 800 to 1050 °C, for 4 min. Then 200 cm^3^/min of H_2_ flow was introduced into the system to remove the water vapour prior to the nanotube growth. Finally, CNT can be grown by flowing 200–300 cm^3^/min of Ar through an ethanol bubbler and 20–50 cm^3^/min of H_2_ for 10 min, followed by cooling under atmospheric H_2_ and Ar, respectively [170].

**Figure 14:**
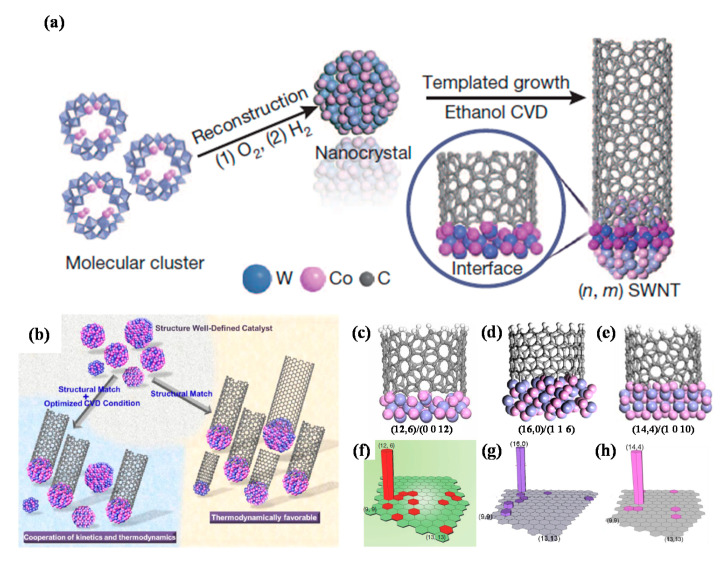
(**a**) Preparation of the W-Co nanocrystal catalysts and the templated growth of a SWCNT with specific chiral indices (*n*, *m*) [168]. (**b**) Schematic illustration of cooperative functions of thermodynamics and growth kinetics [175]. (**c**–**e**) Density functional theory (DFT) simulation of interfaces between the catalyst’s crystal planes and the corresponding SWCNT end structure chirality in side views. (**f**–**h**) SWCNTs chiral maps obtained from Raman measurements that show the relative abundances of various chiralities [168,169,170]. Reproduced with permission from Yang et al., *Nature*, published by Nature, 2014; *Accounts of Chemical Research*, published by American Chemical Society, 2016; *Journal of the American Chemical Society*, American Chemical Society, 2015; and *ACS Nano*, published by American Chemical Society, 2017.

The W_6_Co_7_ alloy nanocatalysts were reduced from W_39_Co_6_O_x_ under different temperature and gaseous phase environments to tune the intermetallic W_6_Co_7_ nanocrystals. Under optimized CVD conditions, the (0 0 12), (1 1 6) and (1 0 10) crystal plane of W_6_Co_7_ individually favors the growth of (12, 6), zigzag (16, 0) and (14, 4) SWCNTs, respectively [168,169,170]. Therefore, as shown in Figure 14, by combining the optimized CVD conditions and the catalyst template effect, proper kinetic and thermodynamic conditions can be achieved for high chirality selective growth of SWCNTs [175].

The synthesis process of CNTs will affect the biological properties as a consequent of different lengths and diameters (aspect ratios) of the produced CNT. It is reported that a high aspect ratio has a detrimental effect on cells [176]. This is due to the fact that cells cannot completely swallow high aspect ratio CNTs, which results in cell damage and the release of harmful oxygen radicals and hydrolytic enzymes [177,178]. Additionally, the presence of residual heavy metals due to the use of metallic catalysts during the synthesis process also affect the biocompatibility of CNTs [179,180].

#### 2.2.3. Tissue Engineering Applications

Reports show that carbon nanotubes are excellent functioning fillers for electro-active scaffolds relevant to a wide range of tissue engineering applications. When in contact with CNTs, cells have been found to become more electrically active, mature and better interconnected. The high aspect ratio of CNTs can structurally simulate certain elongated biomolecules (e.g., for building artificial neural networks or nerve tissue engineering, CNT has the capability of boosting effect on neuron activity, modulating the immune response) useful to mimic the morphology of heart and nerve tissues [181,182,183,184].

**Figure 15:**
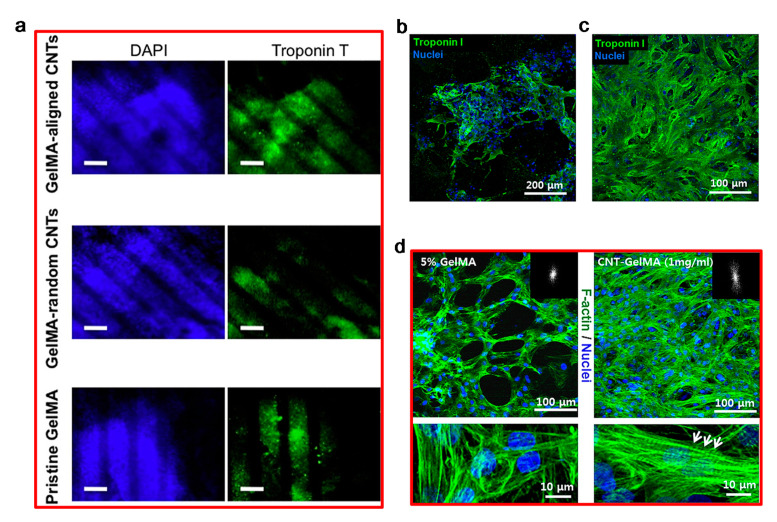
(**a**) Cardiac differentiation analysis by applying DAPI (blue) and Troponin I (green) staining on pure GelMA and CNT–GelMA with embryoid bodies, by Ahadian et al. Low cardiac differentiation is indicated by low expression of Troponin T [185]. (Right) Cardiac cell phenotype examination on the hydrogels by Shin et al. showed more aggregated Troponin I presence on (**b**) GelMA substrates than on (**c**) CNT–GelMA substrates. (**d**) Confocal images of GelMA and 1 mg/mL CNT–GelMA with cardiomyocytes cultured for five days [181]. Reproduced with permission from Ahadian et al., *Acta Biomaterialia*, published by Elsevier, 2016; and Shin et al., *ACS Nano*, published by American Chemical Society, 2013.

For heart tissue engineering applications, Shin et al. and Ahadian et al. [181,185] investigated the use of gelatin methacryloyl (GelMA) hydrogel structures containing CNTs. Using a dielectrophoresis method, Ahadian et al. [185] were able to align the CNTs in GelMA pre-polymer solution (final GelMA concentration of 5% (*w*/*v*)), while Shin et al. [181] produced GelMA containing random CNTs (0, 1, 3 and 5 mg/mL). Although both systems produced positive results in promoting cell differentiation, their behavior under electrical stimulation was. As shown in Figure 15, mouse embryoid bodies (EBs) cultured on the CNTs/GelMA substrate differentiated more toward cardiomyocytes than EBs cultured on pure GelMA and GelMA containing random CNTs. The different impact of electrical stimulation on these substrates (GelMA with no CNTs, randomly dispersed CNTs and aligned CNTs) is because of the higher electrical conductance of CNT containing GelMA in the direction of applied electrical stimulation.

Ho et al. fabricated 3D porous PCL/MWCNT scaffolds with filament distance ranging from 300 to 450 µm with an extrusion-based additive manufacturing system [186]. Authors extensively investigated the effect of adding MWCNT to PCL (1%, 3% and 5% *w*/*w*%) on both mechanical and biological properties of the scaffolds. Nano-indentation studies showed a gradual enhancement in the elastic modulus (increased from 0.51 ± 0.18 GPa for PCL scaffold to 0.87 ± 0.10 GPa for PCL scaffolds containing 5% of MWCNT), hardness (increased from 0.057 ± 0.010 GPa to 0.072 ± 0.003 GPa) and maximum peak load (increased from 1.16 to 1.34 mN). The addition of MWCNTs also contributes to increase the crystallinity level of printed filament, broadening the crystallization peak, due to the restricted mobility of polymer chains in the nanocomposite matrix. MTT assay with H9C2 rat myocardial cells showed no cytotoxic due to the presence of MWCNTs [186]. He et al. also used H9C2 cells to assess PCL–polyethylene oxide (PEO)/MWCNT scaffolds [187]. In this case, scaffolds were fabricated with a fiber diameter of 10 µm, vertical pores of around 800 µm but almost no porosity in the lateral sides, and different concentrations of MWCNT (0, 0.5 and 1.5 *w*/*v*%), using an electrohydrodynamic 3D printing system. The PCL solution was prepared by using acetic acid, and PEO was added to modulate the viscosity. Biological results show that the addition of MWCNT facilitate cell alignment but had a negative effect on cell attachment, due to its agglomeration in the printed fibers, compared to PCL–PEO scaffolds.

**Figure 16:**
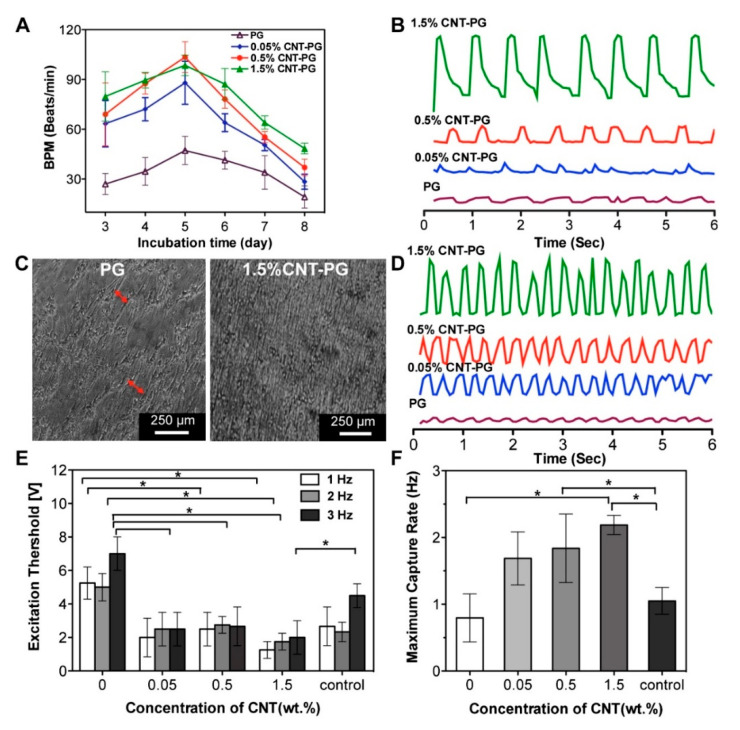
Electrophysiological functions of engineered cardiac constructs. (**A**) Beating frequency (BPM) of constructs as a function of CNT concentration and incubation time. (**B**) Representative spontaneous contraction patterns of CMs cultured on poly(glycerol sebacate):gelatin (PG) scaffolds and CNT incorporated scaffolds recorded after seven days of cultivation. (**C**) Phase-contrast images indicating organized tissue construct and non-continuous aligned tissue (red arrows) on the CNT–PG and PG scaffolds, respectively after seven days of culture. (**D**) Representative contraction patterns of electrically stimulated CMs on PG scaffold compared to CNT–PG scaffold after seven days of culture (frequency = 1). (**E**) Excitation threshold and (**F**) maximum capture rate of CMs seeded on scaffolds, indicating that increasing the CNT concentration and aligned structures significantly reduced excitation threshold and enhanced maximum capture rate (CMs cultured on random 1.5% CNT–PG scaffold was considered as control). (* *p* < 0.05 by one-way ANOVA analysis followed by Tukey’s post-hoc test) [188]. Reproduced with permission from Kharaziha et al., *Biomaterials*, published by Elsevier, 2014.

Kharaziha et al. [188] reported that the addition of CNT into poly(glycerol sebacate):gelatin (PG) nanofibrous scaffolds up to 1.5% could significantly increase the electrical conductivity. As Figure 16 shows, the excitation threshold decreased and both the maximum capture rate and mechanical properties increased by increasing the CNTs content. Fabricated CNT–PG scaffolds also significantly stimulate spontaneous and synchronous beating activity than the compared PG scaffolds [188]. Similarly, Pok et al. [189] demonstrated that the addition of SWCNTs into chitosan-based hydrogel improves the conductivity of the produced scaffold. Results show that scaffolds with SWCNTs concentration lower than 175 ppm did not exhibit any cytotoxicity with neonatal rat ventricular myocytes (NRVM), while scaffolds with concentrations higher than 69 ppm supported the NRVM beat at a consistent rate of 310 beats/min, which is close to rat hearts [189]. Mehdikhani et al. prepared PCL/PEG scaffold samples with and without MWCNTs, using solvent casting and freeze-drying technique. As reported, the conductivity of the scaffold was significantly increased with the addition of MWCNTs, changing from 0.0 S/m (PCL/Polyethylene terephthalate glycol (PETG) scaffold) to 0.45 S/m (fibrin coating containing 1 wt.% MWCNTs). Moreover, fabricated scaffolds are able to sustain viable myoblasts, showing high potential for myocardial tissue engineering [190].

For bone tissue engineering applications, Goncalves et al. produced three-phase interconnected porous scaffolds (hydroxyapatite and CNTs mixed with PCL) with different compositions (50 wt.% PCL, CNTs varying between 0 and 10 wt.%, and hydroxyapatite being the balance) and pore size ranging between 450 and 700 µm, using a pressure-assisted additive manufacturing system [191]. Biological tests were performed by using MG63 osteoblast-like cells. For all compositions, it was possible to observe high cell attachment and proliferation values in scaffolds containing high content of CNTs. Compression tests showed that scaffolds with low CNT content presented larger compressive resistance, while scaffolds with 10% of CNTs were more easily deformed [191]. In another work, a PCL/HA slurry containing ionically modified CNTs (CNT with a positively charged surface) was robotic-dispensed producing scaffolds with pore sizes of around 226 µm. The concentration of HA was set to be 40 *w*/*v*%, and the concentration of CNTs was 0.2 wt.%. Results show that the incorporation of the ionically modified CNTs improved the compressive strength (from 1.5 MPa for PCL scaffolds and 2.0 MPa for PCL/HA scaffolds to 5.5 MPa for PCL/HA/CNT scaffolds) and MC3T3-E1 cell attachment and proliferation. In vivo tests were conducted by implanting the PCL/HA/CNT scaffold into a rat subcutaneous tissue. After four weeks, the results show signs of inflammatory effects due to the presence of the scaffold and the formation of soft fibrous tissue and neo-blood vessels [192].

**Figure 17:**
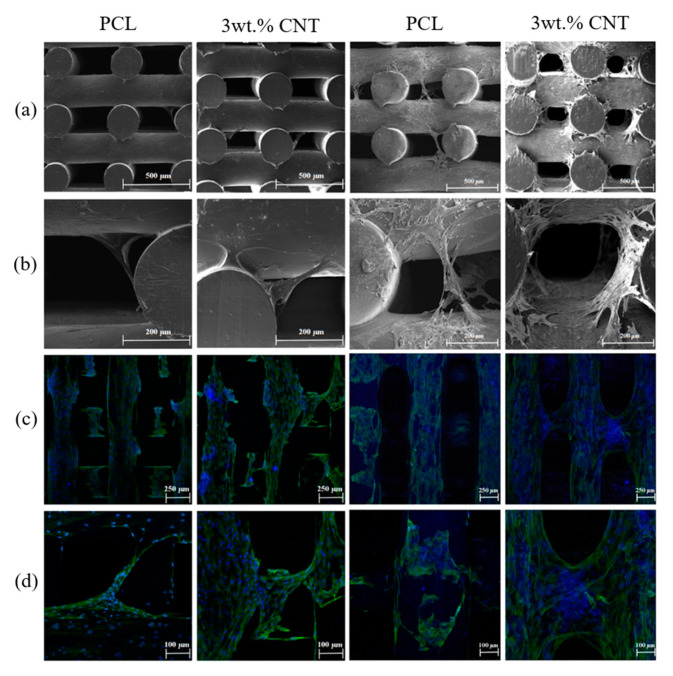
(**a**,**b**) SEM images at 65× *g* and 230× *g* magnification, (**c**,**d**) confocal microscopy images at 100× *g* and 200× *g* magnification of PCL and 3 wt.% PCL/CNT cell-seeded scaffolds, 7 days (left 2 columns) and 14 days (right 2 columns) after cell proliferation [193]. Reproduced with permission from Wang et al., *Journal of the Mechanical Behavior of Biomedical Materials*, published by Elsevier, 2019.

Wang et al. [193] investigated PCL/CNT scaffolds fabricated by extrusion-based additive manufacturing for bone regeneration. With the addition of CNT, the compressive modulus increased from 37.88 ± 1.24 MPa (neat PCL) to 45.47 ± 1.12 MPa (3 wt.% CNT), and the compressive strength increased from 3.18 ± 0.15 MPa (PCL) to 3.83 ± 0.28 MPa (3 wt.% CNT). The water contact angle decreases from 92.62 ± 0.24° (PCL) to 86.18 ± 1.25° (3 wt.% CNT). As shown in Figure 17, in terms of the biological studies, after seven days, the PCL/CNT scaffolds show higher cell affinity and cell proliferation values compared to neat PCL scaffolds [193].

**Figure 18:**
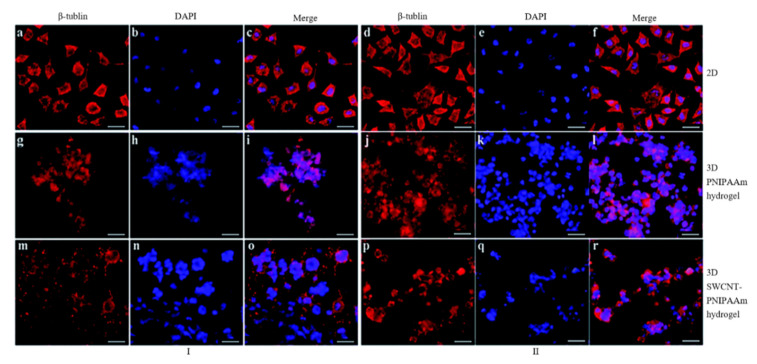
Effects of electrical stimulation (I) on SH-SY5Y cell morphology compared to a group with no applied electrical stimulation (II) (scale bar ¼ 40 mm). No significant difference in 2D groups (**a**–**f**) and 3D PNIPAAm hydrogel groups (**g**–**l**) in cell morphology. However, neurite outgrowth was enhanced on SWCNT–PNIPAAm group with electrical stimulation (**m**–**o**) compared with no electrical stimulation (**p**–**r**) [194]. Reproduced with permission from Sang et al., *RSC Advances*, published by Royal Society of Chemistry, 2016.

For nerve tissue engineering applications, electro-active scaffolds containing CNTs have been produced by using different polymers and hydrogels. Sang et al. produced single-walled carbon nanotube–poly(n-isopropylacrylamide) (SWCNT–PNIPAAm) structures through copolymerization of n-isopropylacrylamide and single-walled carbon nanotubes [194]. The effects of electrical stimulation on the morphology of SH-SY5Y cells on 2D culture, PNIPAAm hydrogel 3D culture and SWCNT–PNIPAAm hydrogel 3D culture were investigated. From Figure 18, it is possible to observe that neurite outgrowth is more apparent by using 3D SWCNT–PNIPAAm hydrogel subjected to electrical stimulation. As observed, electrical stimulation can significantly increase neurite sprouting, gather the dividing cells and form multinucleate cells. The effect of the electrical conductivity of the SWNT–PNIPAAm hydrogel on SH-SY5Y cells was confirmed through a significant increase in neurite number and largely enhanced neurite outgrowth.

**Figure 19:**
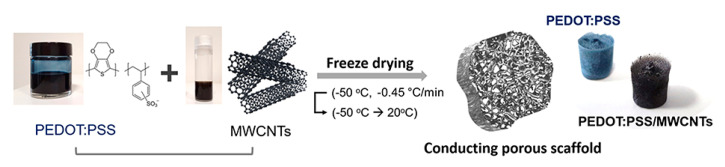
The components and process used for the fabrication of 3D conducting scaffolds. The photos show free-standing conducting scaffolds based on PEDOT:PSS and PEDOT:PSS/ multi-walled carbon nanotubes (MWCNTs) [196]. Reproduced with permission from Jayaram et al., *Frontiers in Chemistry*; published by Frontiers Media S.A., 2019.

For other applications, Dominguez-Alfaro et al. [195] fabricated porous PEDOT/CNT scaffolds through a vapour phase polymerization method. Results show that the impedance of PEDOT/CNT (|*Z*_PEDOT/CNT_| = 6 kΩ) scaffolds at 0.1 Hz was significantly lower than PDMS/CNT (|*Z*_PDMS/CNT_| = 50 kΩ) and naked electrode filled with electrolyte PBS solution (|*Z*_PBS_| = 90 kΩ). The fabricated scaffolds also show good biocompatibility with mouse astrocytes C8-D1A cells and have a positive effect on promoting cell growth in the first three days [195]. Jayaram et al. produced 3D hybrid poly(styrene sulfonate) (PSS)/MWCNT composite scaffolds, using a freeze-drying method as shown in Figure 19, results show that the resistivity of the scaffolds containing MWCNT was 7 times lower than the scaffolds without MWCNT [196].

Other techniques like freeze-casting and electrospinning were also used to produce different types of polymer/CNT polymer/ceramic/CNT and bioglass/CNT scaffolds [197,198,199,200,201,202,203]. In all cases, results show that the presence of CNTs improved compression strength and elastic modulus and had a positive effect on the biological performance (cell attachment, proliferation and differentiation) of the scaffolds. Additional reported studies using CNTs are summarized in Table 3.

Despite the successful use of CNTs as reinforcements of a wide range of polymer based scaffolds and substrates, the biocompatibility and biosafety of CNTs still require further investigation [159]. It has been reported that CNTs with high aspect ratios show toxicity similar to asbestos fibers [219], potentially inducing inflammation and fibrosis [220,221]. In addition, the surface functional groups attached to CNTs can change the interaction with the cell membranes and further control the penetration of CNTs into the cells [222,223]. Moreover, the catalyst particles may affect the biosafety because they can introduce oxidative stresses, cross cell membranes and generate free radicals [224]. Therefore, changing critical parameters such as size, impurities, surface chemistry, surface charge, reactivity, morphology and crystal structure can significantly influence the toxicity of CNTs [96]. In addition, there is a huge difference in toxicity patterns of CNTs in comparison to graphene and GO, due to the differences in their synthesis route and structural morphology [225].

## 3. Conclusions and Future Perspectives

The field of tissue engineering is experienced exciting advances toward the fabrication of smart and biomimetic constructs as alternatives to current clinical therapies. These advances strongly rely on the use of advanced materials and new fabrication techniques. Electrospinning and additive manufacturing have been successfully explored to produce scaffolds for skin, bone, nerve and muscle regeneration. Usually, these techniques use single material (polymers, hydrogels, ceramics and composites) to produce cell substrates designed according to specific requirements like porosity, mechanical properties, surface properties and degradation characteristics. Recent studies showed the relevance of using materials to stimulate cells increasing cell attachment, proliferation and differentiation. Electrically conductive materials, as discussed in this paper, could have a significant impact in tissue engineering, as there is an evidence that electrical stimulation is useful for stimulate-guided growth of cells.

Scaffolds made with different polymers reinforced with a wide range of electrically conductive materials have been proposed and assessed from a biological point of view. As discussed in this review, the exceptional electrical and surface properties of CNMs, especially graphene and CNTs, together with their controllable morphologies make them important components for the development of novel electro-active scaffolds. A wide range of 2D membranes were initially produced and assessed with different cell lines. Recently, the development of additive manufacturing enabled the use of CNMs blended with polymers to create 3D porous scaffolds in a controlled and reproducible way. Through additive manufacturing, it will also be possible to create complex multi-material and functional gradient scaffolds, containing different regions with and without electrically conductive fillers. Functional gradient scaffolds like these can be relevant to produce tissue interfaces, using hADSCs, where the elastic and flexible nature of CNMs not only improves the mechanical properties of the scaffolds, but also synergize the effects of electrical stimulation on both cell proliferation and differentiation. However, very few papers compare the performance of different CNMs. Wang et al. reported that graphene filler presents better chemical and physical properties than CNT under the same amount [193], while Srikanth et al. also reported that graphene has greater toxicity than CNT [176].

Different strategies to incorporate CNMs have been explored. The majority of the studies, as reported in this review, used chemical or physical blending approaches. However, in this case, techniques such as additive manufacturing or electrospinning cannot allow the fabrication of structures with high levels of CNMs due to rheological constrains. However, some researchers also explored new routes to create core–shell structures with CNMs as the outer layer and a polymeric material as the core [226]. This approach has the advantage of allowing to obtain structures with high surface CNMs concentration, significantly improving the electrical conductivity. However, through this approach, the CNMs will not significantly contribute to the overall improvement of mechanical properties, considering the long-term use of these structures, and may pose significant cytotoxicity problems, compromising the biological performance of the structures.

This review mainly focuses on the biological performance of electro-active structures. However, several research works also demonstrated the antimicrobial properties of polymer/CNM scaffolds. CNMs such as graphene have been identified to have antibactericidal activity on various bacteria due to their sharp edges and oxidative stress induction. This should be further explored, for example to create new tissue engineering scaffolds with improved biological and antibacterial properties. Currently, the design of scaffolds for tissue engineering applications neglects the important issue of bacterial infection of the damaged tissue.

As presented in this review, electro-active scaffolds containing small amounts of CNMs were successfully used for tissue regeneration. However, a possible alternative route, not fully explored in this paper, is the fabrication of structures containing a very high concentration of CNMs, not to stimulate cell proliferation and differentiation, but cell apoptosis, targeting cancer treatment application.

Current major challenges are related to the lack of comprehensive in vivo studies for adequate assessment of long-term effects in terms of biocompatibility and cytotoxicity. The critical cytotoxicity concentration of these materials for different cell types is not yet clear. Their cellular and biological interactions, especially relating to cellular uptake mechanisms and biocorona formation, is not fully understood either. These require further investigations, conducting systematic comparative studies which will differentiate results of humans from animals.

Challenges regarding the synthesis of CNMs with controlled size, shapes and functionalities should also be addressed as it affects the overall biocompatibility and performance of CNM-based electro-active structures. In the case of electro-active porous scaffolds, a key parameter is the control of its degradability, which must match the regeneration rate of the new tissue. However, the in vivo biodegradability of the electrically conductive carbon nanomaterials is still not fully investigated, representing an important research challenge. In-depth in vivo experiments for better understanding the interaction between electro-active structures and the surrounding tissues and their performance toward tissue regeneration are still required.

## Figures and Tables

**Table 1:** Additive manufacturing techniques.

Methods	Working Principle
Material extrusion	An additive manufacturing process in which polymers or polymer-based composites in the form of pellets or filaments are melted and selectively dispensed trough a nozzle or orifice
Material jetting	Polymeric droplets or bioinks (hydrogels containing cells and growth factors) are selectively deposited
Binder jetting	An additive manufacturing process in which a liquid binding material (e.g., colloidal system) is selectively deposited to join powder materials
Vat photopolymerization	An additive manufacturing process in which a liquid photopolymer is polymerized or cured (transition from liquid to solid), using a light source (laser or lamp)
Powder bed fusion	An additive manufacturing technique in which thermal energy from a laser or an electron beam is used to fuse in a selective way material in a powder form
Directed energy deposition	A technique in which focused thermal energy is used to fuse materials as the material is being deposited
Sheet lamination	Sheets of materials (e.g., paper, polymers, ceramics and metals) are cut and bonded together, to form a 3D object

**Table 2:** Studies using graphene electro-active structures.

Electro-Active Structures	Electrical Stimulation Settings	Cell Line	Outcome	Reference
Cellulose/graphene scaffold	100 mV/mm of DC for 1 h/day	Human adipose stem cells	Increased proliferation, mineral deposition and ALP expression	Li et al., 2020 [123]
Reduced graphene oxide-coated ApF/poly(l-lactide-co-ε-caprolactone) scaffold	100 mV/cm for 1 h/day	SCs and PC12 cells	Promoted SC migration, proliferation, myelin gene expression, neurotrophin secretion and induced PC12 cell differentiation	Wang et al., 2019 [124]
Polypyrrole/graphene nanofibrous scaffold	Forward potential varied from 0.1 to 1 V/cm while reverse potential changed from −0.1 to −1 V/cm	Retinal ganglion cells	led to 137% improvement in cell length with a significantly enhanced antiaging effect for RGCs	Yan et al., 2016 [125]
Graphene scaffold	Square waveform with 1 Hz and 10 μA for 30 min/day	Human Rett-derived neuronal progenitor cells	Improved cell maturation	Nguyen et al., 2018 [126]
Graphene membrane	Intensity of 100 mV/mm with 1 ms duration at 10 or 1 Hz	PC-12 nerve Cell	Promoted neurite extension and length growth	Meng et al., 2014 [127]
Graphene membrane	Pulse of 15 V, duration 50–100 ms	C2C12 Myoblasts	High degree of myogenic differentiation	Bajaj et al., 2014 [128]
Bacterial Cellulose/Poly(3,4-ethylenedioxythiophene) (PEDOT)/GO membrane	0.5 V cm^−1^ for 1–100 ms lower than 0.6 V	PC12 neural cells	Promoted cell orientation and development of PC12 cells	Chen et al., 2016 [129]
Graphene-based membrane	8 V at 1 Hz with 10 ms duration	Mouse C2C12 myoblast cells	Enhanced differentiation of skeletal muscle cells	Ahadian et al., 2014 [130]
Methoxy PEG/rGO membrane	1–100 ms monophasic anodic pulses, 10 s duration, 0.6 V pulse potential	PC12 neural cells	Predominant increase in cell percentage with higher action potentials	Zhang et al., 2014 [131]
Poly(lactic-co-glycolic acid) (PLGA)/GO membrane	100 mV at 20, 100 and 500 Hz for 1 h/day	Neural stem cells	Promoted proliferation, differentiation and neurite elongation in NSCs	Fu et al., 2019 [132]
Rolled GO foam	100 ms cathodic voltage pulses	Human neural stem cells	More proliferation of hNSCs and their accelerated differentiation into neurons	Akhavan et al., 2016 [133]
Graphene-based foam	−0.2–0.8 V, 1–100 ms monophasic cathodic pulses at 10 s intervals, 20–30 μA threshold	Neural stem cell	Supported cell growth and enhanced differentiation to neurons than astrocytes	Li et al., 2013 [134]
Graphene-based substrate	0.3 V at 1 Hz	Human mesenchymal stem cells	Did not create a cytotoxic environment	Balikov et al., 2016 [135]
Graphene-based substrate	100 mV at 50 Hz for 10 min/day	Mesenchymal stem cells	Transdifferentiation of MSCs to SC-like phenotypes solely without the need for additional chemical growth factors	Das et al., 2017 [136]
Graphene/polyacrylamide hydrogel membrane	5 V with 10 ms duration at 1 Hz for 4 h/day	Mouse C2C12 myoblast cells	Increased myogenic gene expression levels of myoblasts	Jo et al., 2017 [137]
CS/oxidized hydroxyethyl cellulose/rGO/asiaticoside liposome-based hydrogel membrane	250 mV for 8 h	RSC 96 cells, PC12 cells, NIH/3 T3 cells	Promoted nerve regeneration	Zheng et al., 2019 [138]
Graphene crosslinked collagen cryogel membrane	1 V for 5 min at 0.20 V/mm	BM-MSCs	Promoted proliferation of cells, aiding neural connections establishment, increase immune-modulatory secretions	Agarwal et al., 2021 [139]

**Table 3:** Studies using CNT electro-active structures.

Electro-Active Structures	Electrical Stimulation Settings	Cell Line	Outcome	Reference
Polylactic acid (PLA)/MWCNT scaffold	DC: 100 μA (4 h/day, 6 days)	Osteoblasts	Proliferation and elongation along the current direction	Shao et al., 2011 [183]
PEGDA/MWCNT scaffold	100, 500 and 1000 µA at 100 Hz for 100 µs	Neural stem cells	Higher TUJ1 and GFAP expression	Lee et al., 2018 [204]
PLGA/MWCNT scaffold	40 mV rectangular pulse for 30 min	PC12 and Schwann cells	Promoted the growth and myelination of Schwann cells	Wang et al., 2018 [205]
PCL/CNT scaffold	5 V cm^−1^ for 5 ms duration at 1 Hz every 4 days	Human Mesenchymal Stem Cells	Rapid morphological changes and expressed cardiac genes	Crowder et al., 2013 [184]
124 polymer/CNT scaffold	2-ms pulses of 0–0.1 V at 1 Hz	Neonatal rat heart tissue	Improved tissue maturity	Ahadian et al., 2017 [206]
Polyvinyl acetate/Chitosan/CNT scaffold	5 mV·cm^−^^1^ in a frequency of 1Hz for 5days at 37 °C	Undifferentiated mesenchymal stem cells	Enhanced the adherence of MSCs	Mombini et al., 2019 [207]
PLA/CNT nanofiber scaffold	0.15 V/cm for 2 ms duration at 1 Hz	Mesenchymal stem cell	Increased protein expression of cardiac-associated markers	Mooney et al., 2012 [208]
ssDNA bound CNT scaffold	0, 50, 100, 200, 300 and 600 mV/mm at 20 Hz	MC3T3 pre-osteoblast cells	Robust cellular filaments and strong focal adhesions sites around cell edges	Liu et al., 2020 [209]
Polycaprolactone fumarate/CNT scaffold	100 mV mm^−1^ at 20 Hz for 2 h/day	PC-12 cell	Enhanced cell proliferation, cell migration and formation of intracellular connections	Zhou et al., 2018 [210]
Phosphate glass microfibers/CNT scaffold	5 mA at 1 Hz for 1 ms duration	PC12 and DRG cells	Can support nerve regeneration	Ahn et al., 2015 [211]
PCL/CNT scaffold	55 ± 8 mV cm^−1^ at 60 Hz for 30 min/day	Osteoblast-like cells (MG63)	Promoted bone mineralization	Jin et al., 2013 [212]
MWCNT scaffold	200 μs pulses of 1–50 V at 40 s intervals	Neurons	Neurite regrowth in spinal explants is favored	Alessandra et al., 2012 [213]
Regenerated bacterial cellulose/polypyrrole/CNT hydrogel membrane	10 μA for 60 min/day	Mouse embryo fibroblast	Improved cell proliferation	Wang et al., 2019 [214]
Poly-L-lactide/CNT substrate	AC: 10 mA (10 Hz, 6 h/day)	Osteoblasts	46% increase in cell proliferation after 2 days	Supronowicz et al., 2002 [215]
PEDOT/CNT substrate	−0.9–0.5 V at scan rate of 100 mV s^−1^ followed by 0.30 mC cm^−2^ at 50 Hz	NB-39-Nu human Neuroblastoma	Higher cell proliferation and longer neurite lengths	Depan and Misra, 2014 [216]
PCL/CNT membrane	750 mV, 100 Hz AC for 30 min daily for 3 to 6 days	PC12 cells	Induced neural differentiation	Su and Shih, 2015 [217]
Nerve growth factor/collagen/CNT membrane	500 mV, using Ag/AgCl electrodes	PC 12 cells	Massive release of NGF consequently supporting neurite sprouting and growth	Cho and Borgens, 2013 [218]

MWCNT, multi-walled carbon nanotube.
